# Supplementary material for: A deep learning-based predictive simulator for the optimization of ultrashort pulse laser drilling
Source: Commun Eng. 2023 Jan 7;2:1. doi: 10.1038/s44172-022-00048-x (PMC10956000; doi:10.1038/s44172-022-00048-x)
Supplement: Supplementary file 2 — Supplementary Information [file 44172_2022_48_MOESM2_ESM.pdf]

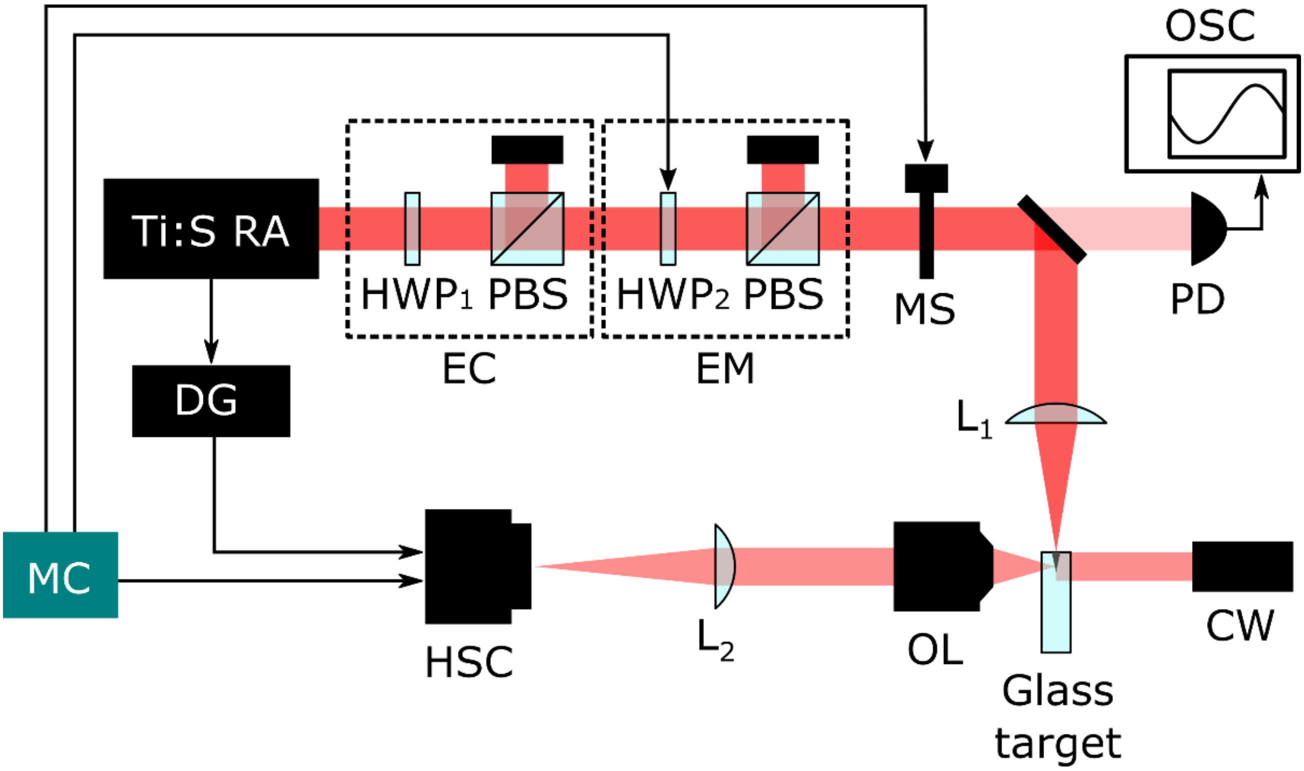

Supplementary Fig. 1 Diagram of experimental setup. Ti:S RA: Regeneratively amplified Ti:Sapphire, DG: Delay Generator. MC: Microcomputer board, HSC: High-speed camera, OL: 20x Objective Lens, CW: Continuous-wave 637 nm laser diode, EC: Manual pulse energy controller, EM: Dynamic pulse energy modulator, HWP_1: Half waveplate mounted on manual rotational stage, HWP_2: Half waveplate mounted on an electronic rotational stage, PBS: Polarization beam splitter, MS: Mechanical Shutter, PD: Photodetector, OSC: Oscilloscope, L_1: f=150 mm plano-convex lens, L_2: f=300 mm plano-convex lens.

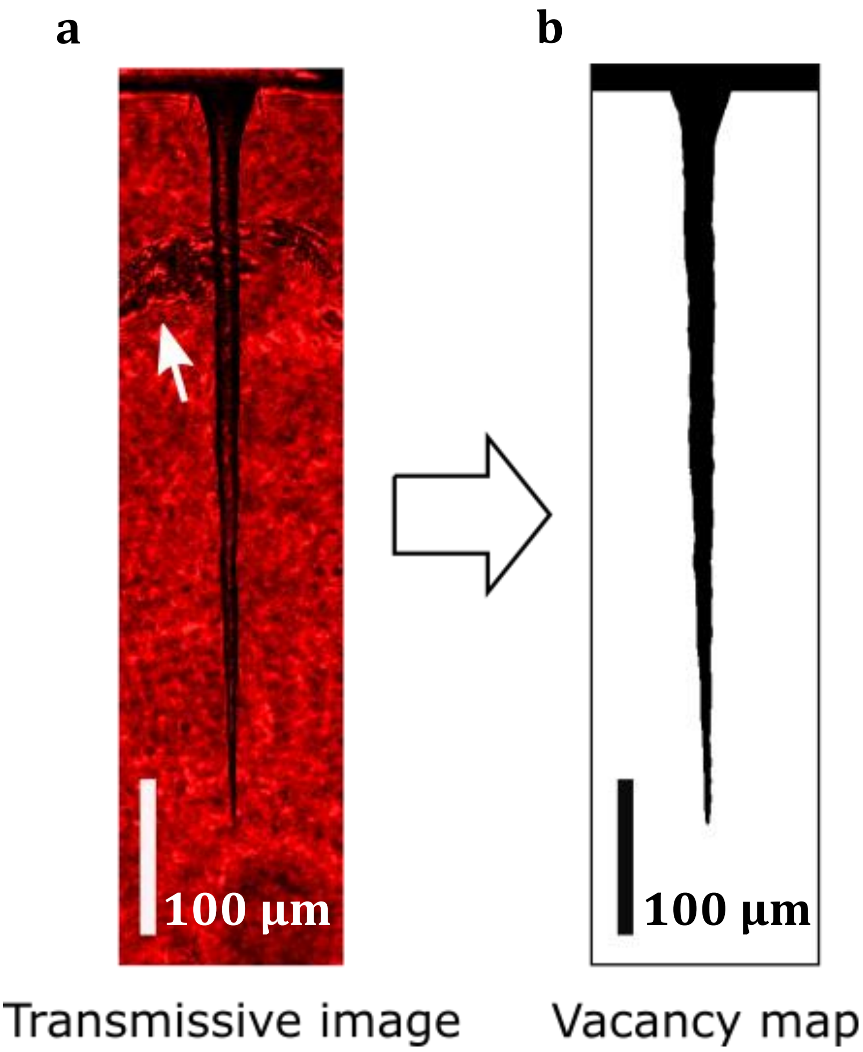

Supplementary Fig. 2 Example of a transmissive image of a microchannel (a) converted to a vacancy map (b). The white arrow points to a damage formed on the side surface of the glass sheet during the drilling process (See also Supplementary Fig. 3). In the vacancy map, such damage as well as interference patterns caused by reflection between optical components are removed. Scale bars are 100 μm.

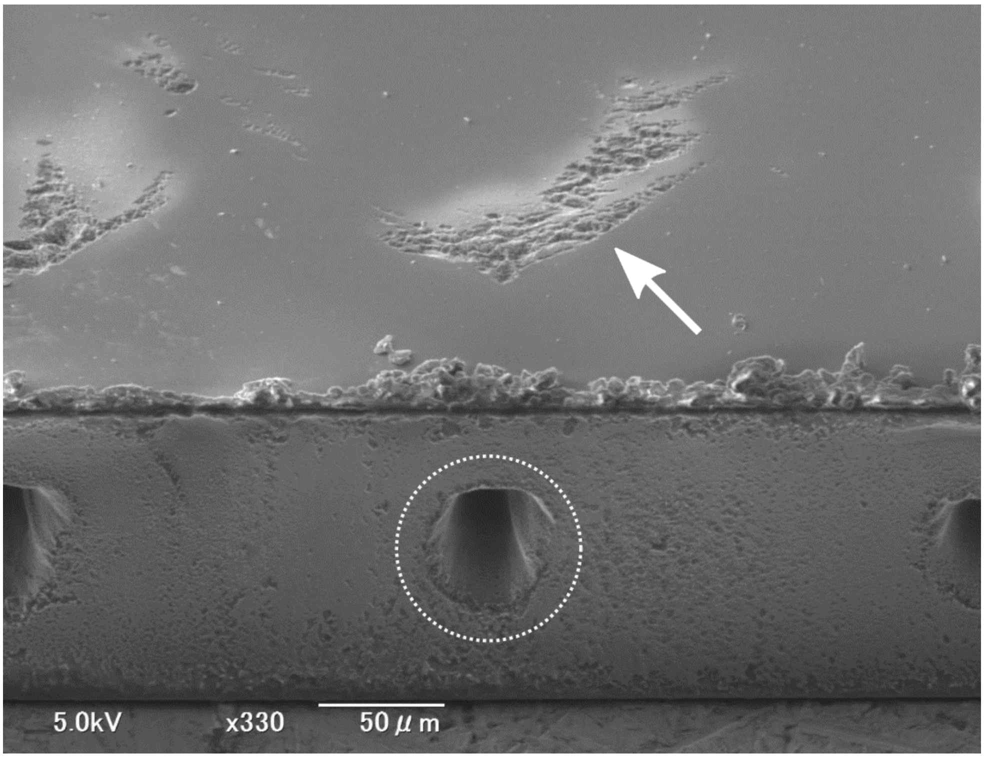

Supplementary Fig. 3 SEM image of the top view of a microchannel. The image was captured with a SEM arm of a multibeam system (JIB-4700F, JEOL) from a 53° angle from the horizontal plane. The white arrow points to the damage created on the surface of the glass sheet during the drilling process. The white dotted circle shows the entrance of the microchannel.

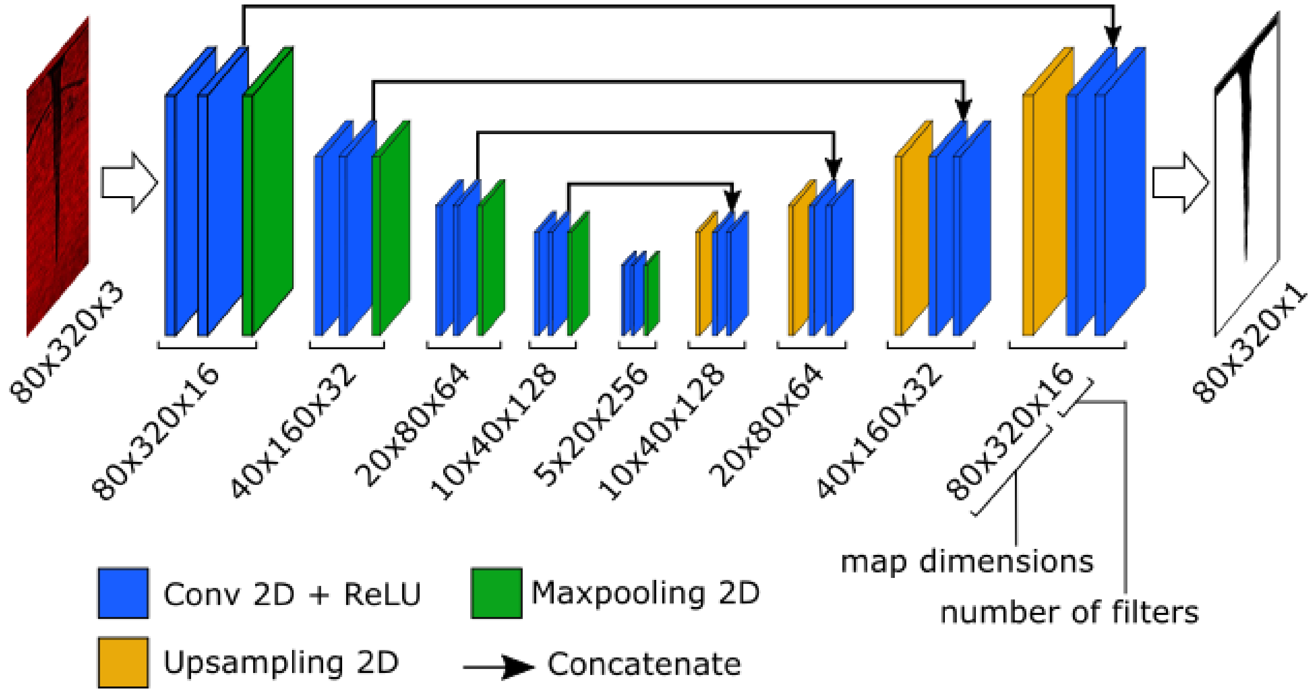

Supplementary Fig. 4 Structure of DNN for vacancy map generation. Conv 2D operations (blue layers) were conducted with a kernel size of 3 and a stride of 1. Maxpooling 2D (green layers) operations were conducted with a pooling size of 2. Upsampling 2D operations (yellow layers) were conducted with a kernel size of 3 and a stride of 2. Concatenation operations (bold arrows) were conducted by merging two outputs along the filter axis.

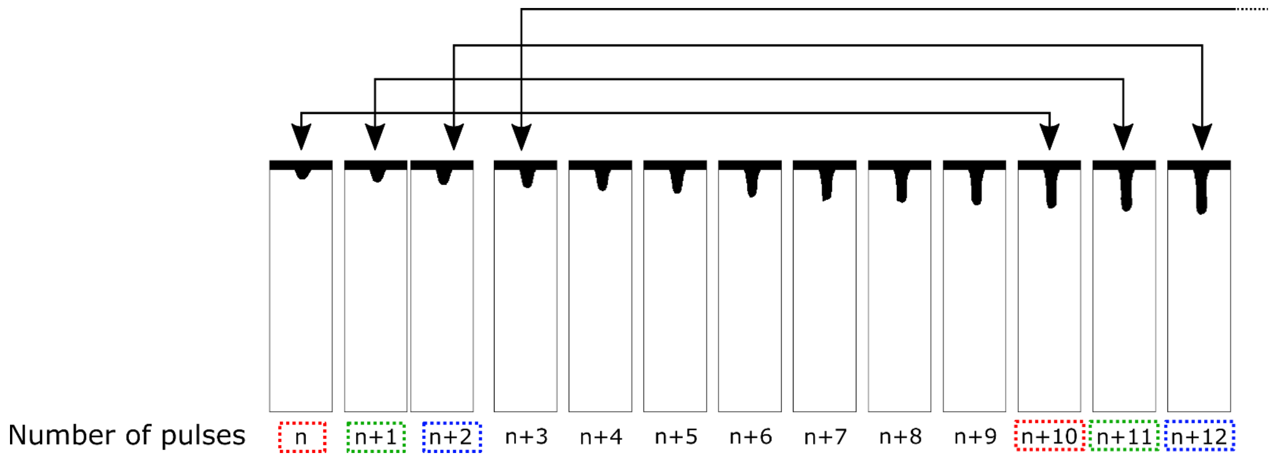

Supplementary Fig. 5 Diagram of pair extraction from a series of vacancy maps. Vacancy maps 10 irradiates pulses apart (images connected by double-sided arrows) were extracted to form an input-output pair for training the DNN simulator.

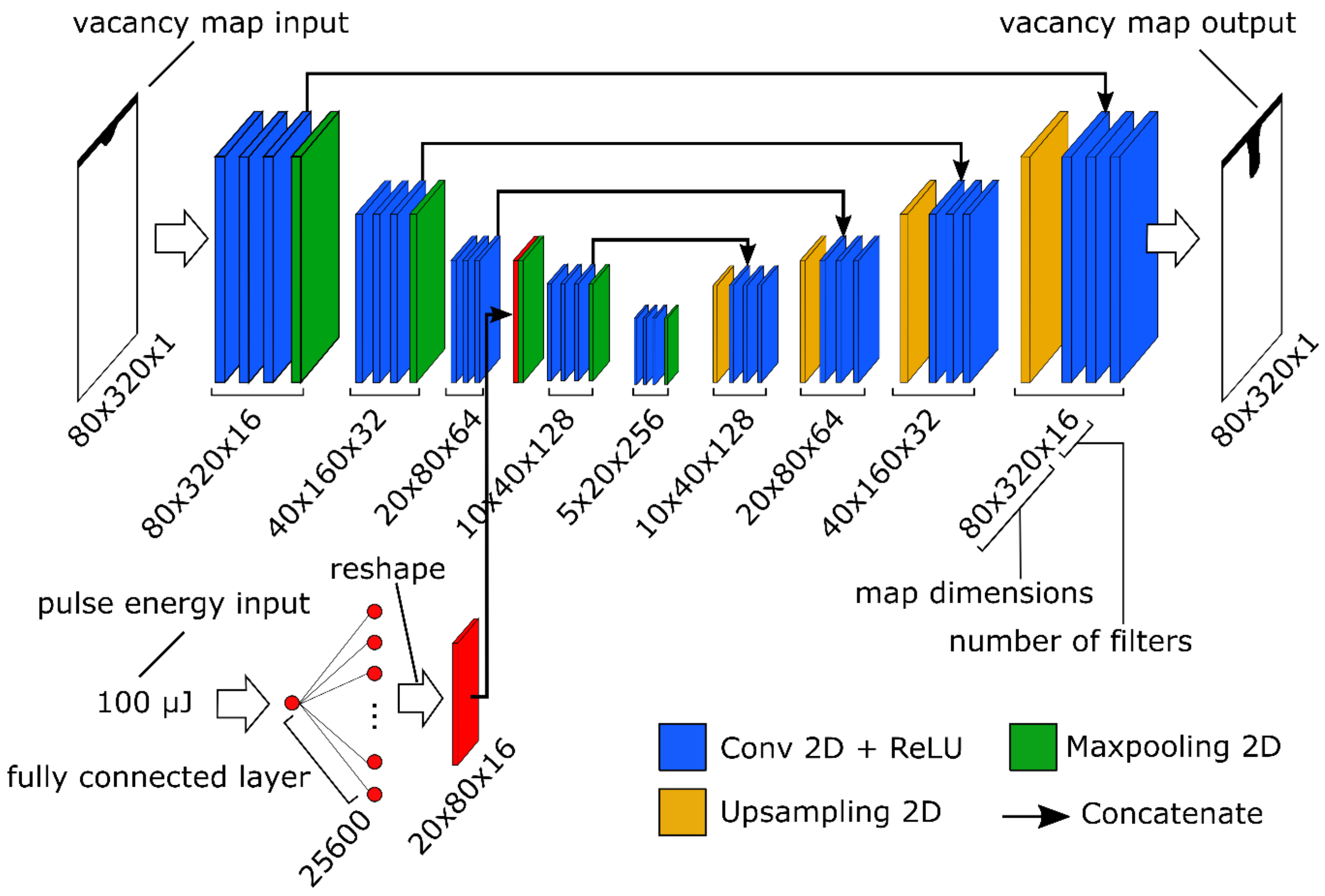

Supplementary Fig. 6 Structure of the DNN simulator. Conv 2D operations (blue layers) were conducted with a kernel size of 3 and a stride of 1. Maxpooling 2D (green layers) operations were conducted with a pooling size of 2. Upsampling 2D operations (yellow layers) were conducted with a kernel size of 3 and a stride of 2. Concatenation operations (bold arrows) were conducted by merging two outputs along the filter axis. In the pulse energy input branch (red layers), a single perceptron taking a pulse energy value is connected to 25,600 perceptrons via a fully connected layer with trainable weights, and the output of those perceptrons are reshaped into a map with dimensions of 20x80 and filter depth of 16, to be merged to an intermediate output of the U-Net structure.

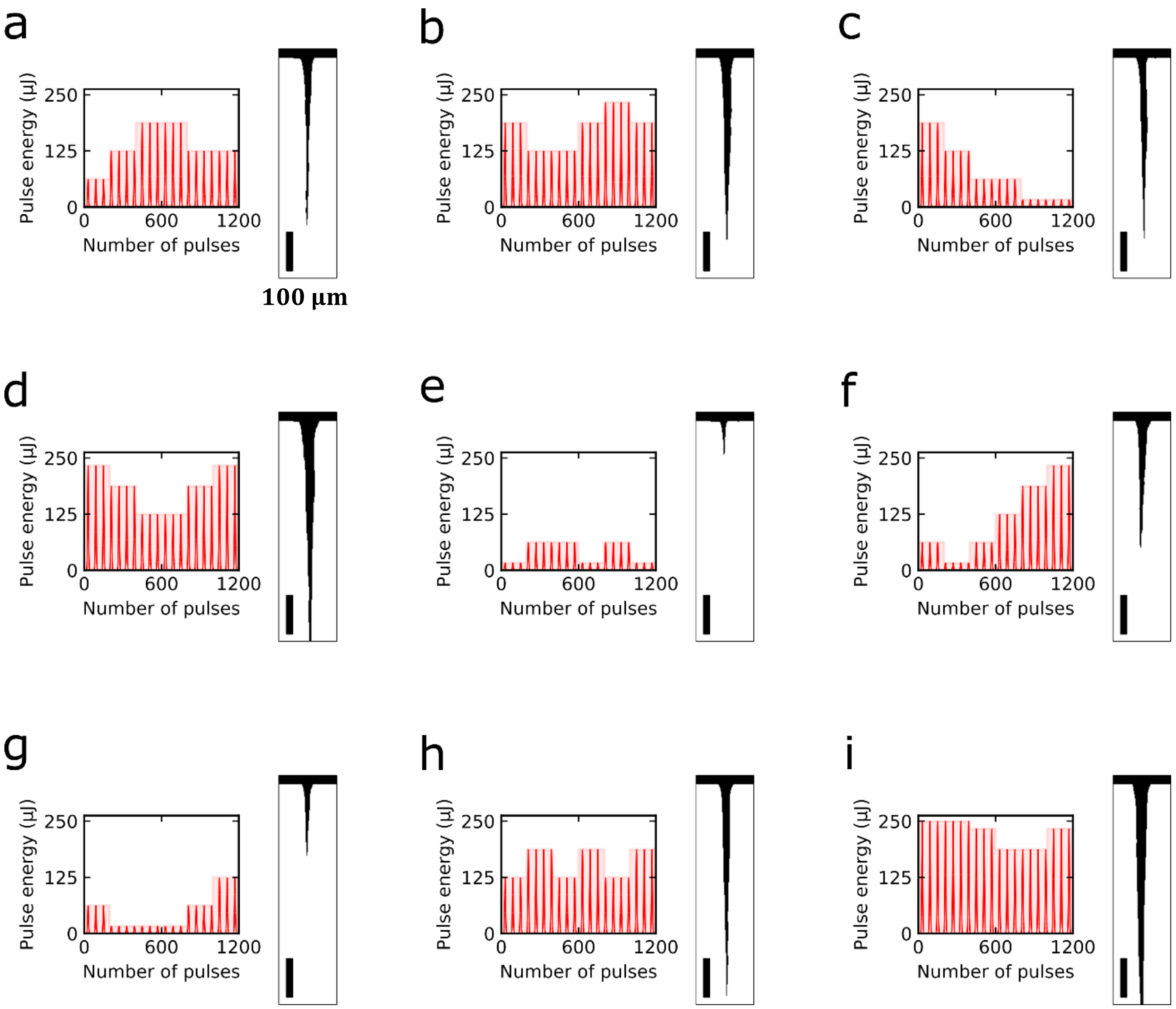

Supplementary Fig. 7 Examples of simulation results from grid simulation. a-i Plot of energy modulations (left) and the corresponding simulation result (right). Scale bars are 100 μm.

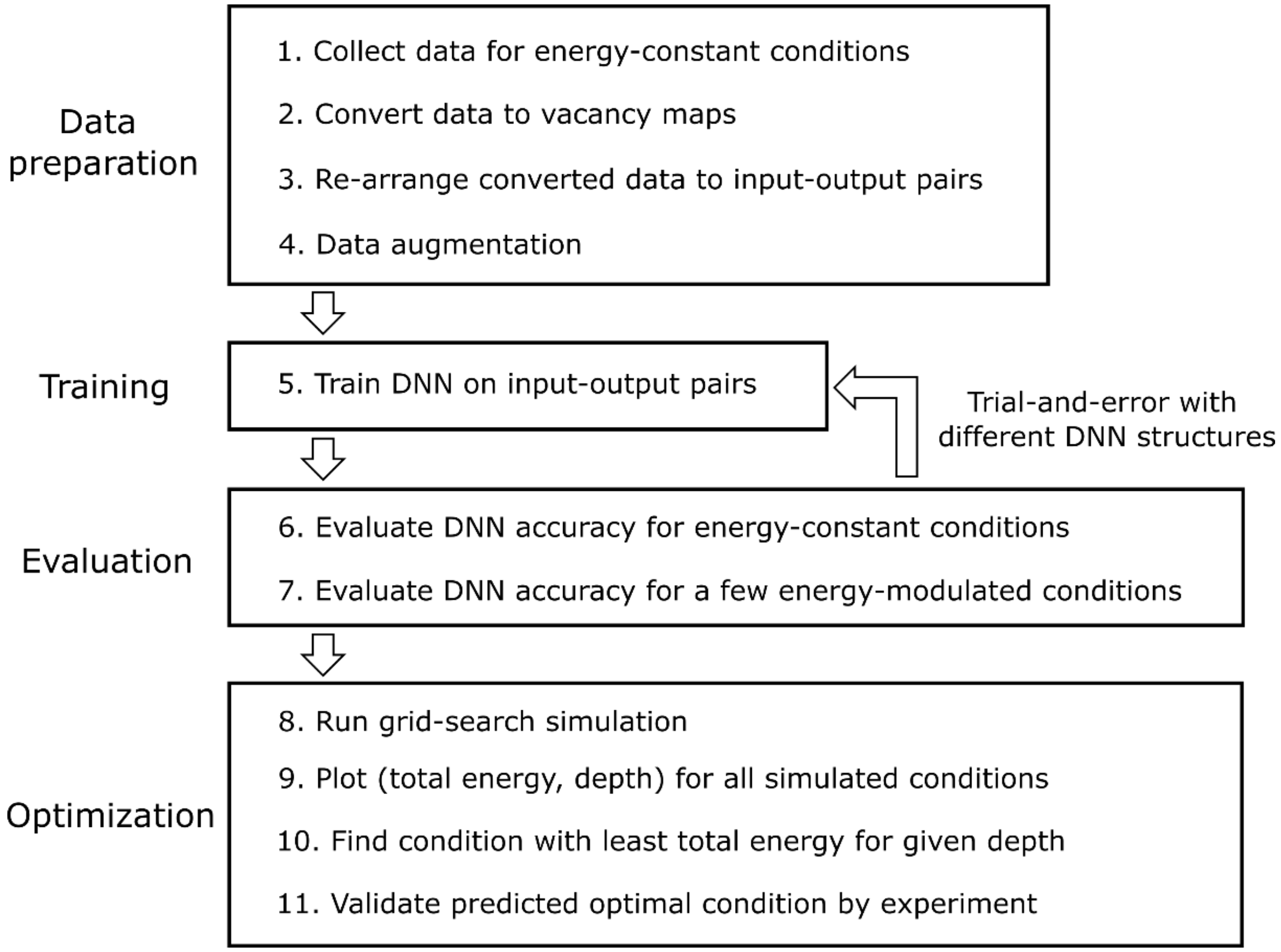

Supplementary Fig. 8 Flow chart of the operations conducted in this work.

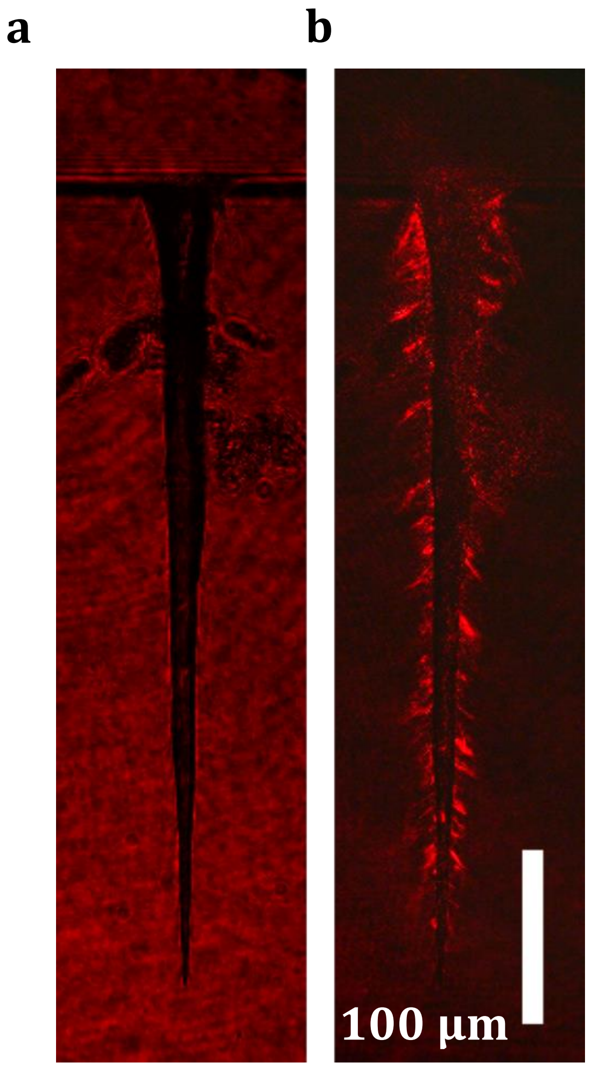

Supplementary Figure 9. Comparison of a transmissive image (a) and polarization image (b) of a typical microchannel. Microscopic cracks appear as bright whiskers in the polarization image. Scale bar is 100 μm.
