# Supplementary material for: A deep learning-based predictive simulator for the optimization of ultrashort pulse laser drilling
Source: Commun Eng. 2023 Jan 7;2:1. doi: 10.1038/s44172-022-00048-x (PMC10956000; doi:10.1038/s44172-022-00048-x)
Supplement: Supplementary file 3 — Description of Additional Supplementary Files [file 44172_2022_48_MOESM3_ESM.pdf]

Description of Additional Supplementary Files

File name: Supplementary Movie 1

Description: The simulation result (left) and corresponding experimental data (right) for a drilling process with constant pulse energy of 50 µJ.

File name: Supplementary Movie 2

Description: The simulation result (left) and corresponding experimental data (right) for a drilling process with constant pulse energy of 150 µJ.

File name: Supplementary Movie 3

Description: The simulation result (left) and corresponding experimental data (right) for a drilling process with constant pulse energy of 250 µJ.

File name: Supplementary Movie 4

Description: The simulation result (left) and corresponding experimental data (right) for the optimal drilling condition predicted by the neural network simulator.

File name: Supplementary Data 1

Description: Data points plotted in Figure 2d.

File name: Supplementary Data 2

Description: Data points plotted in Figure 4a.
