# Supplementary figures and images for: A deep learning-based predictive simulator for the optimization of ultrashort pulse laser drilling
Source: Commun Eng. 2023 Jan 7;2:1. doi: 10.1038/s44172-022-00048-x (PMC10956000; doi:10.1038/s44172-022-00048-x)

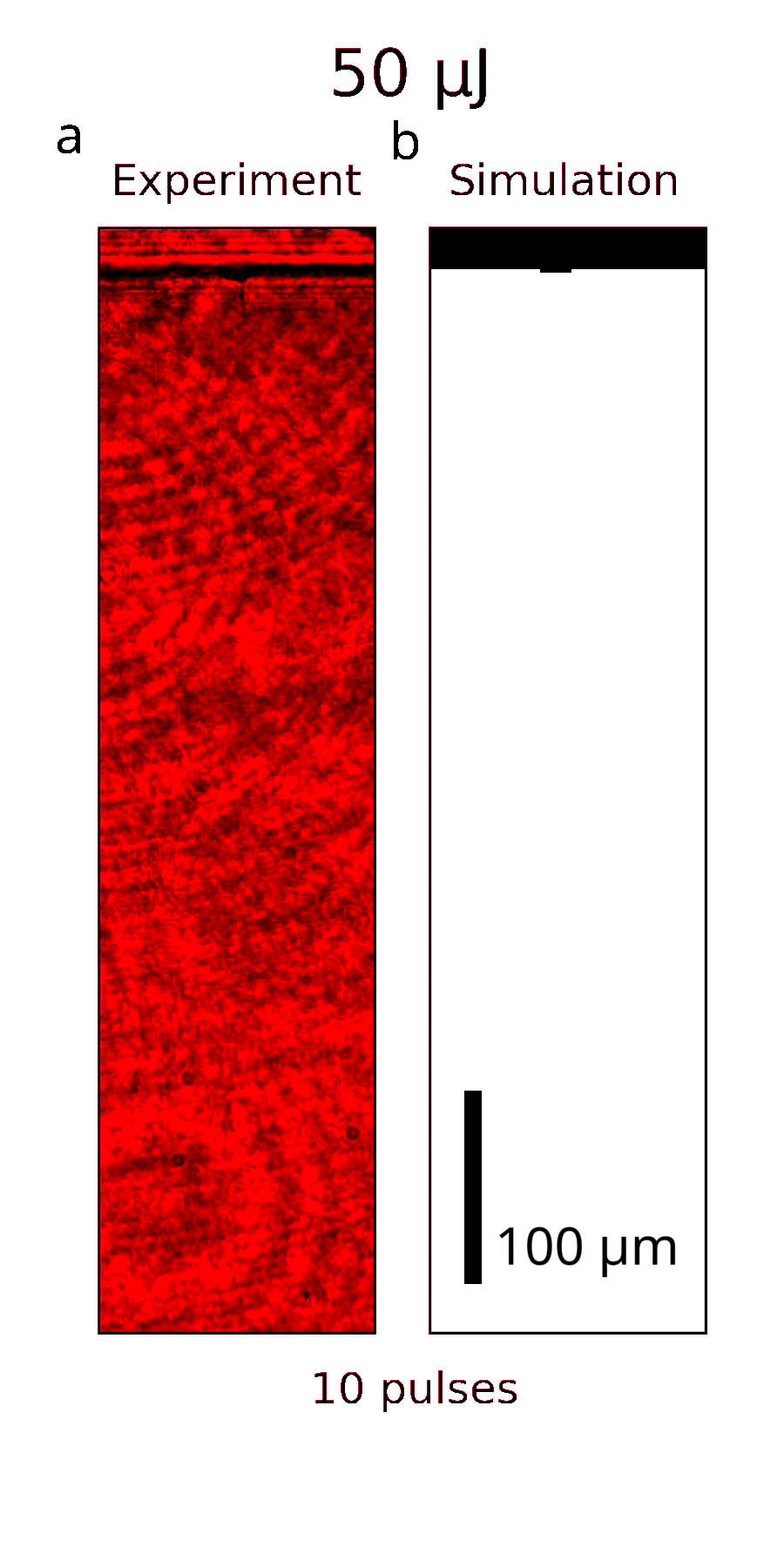

Supplement: Supplementary file 4 — Supplementary Movie 1 [file 44172_2022_48_MOESM4_ESM.gif]

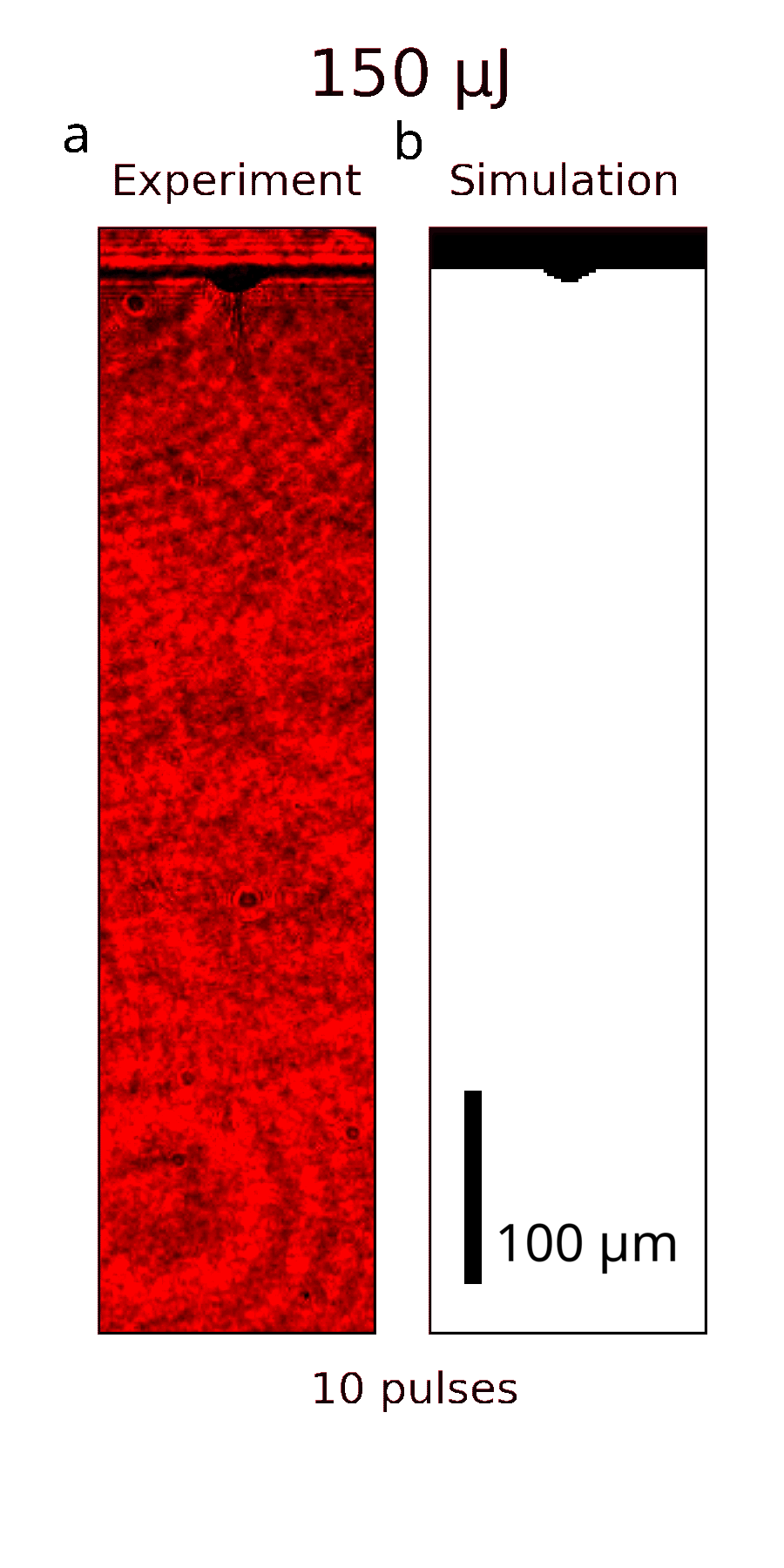

Supplement: Supplementary file 5 — Supplementary Movie 2 [file 44172_2022_48_MOESM5_ESM.gif]

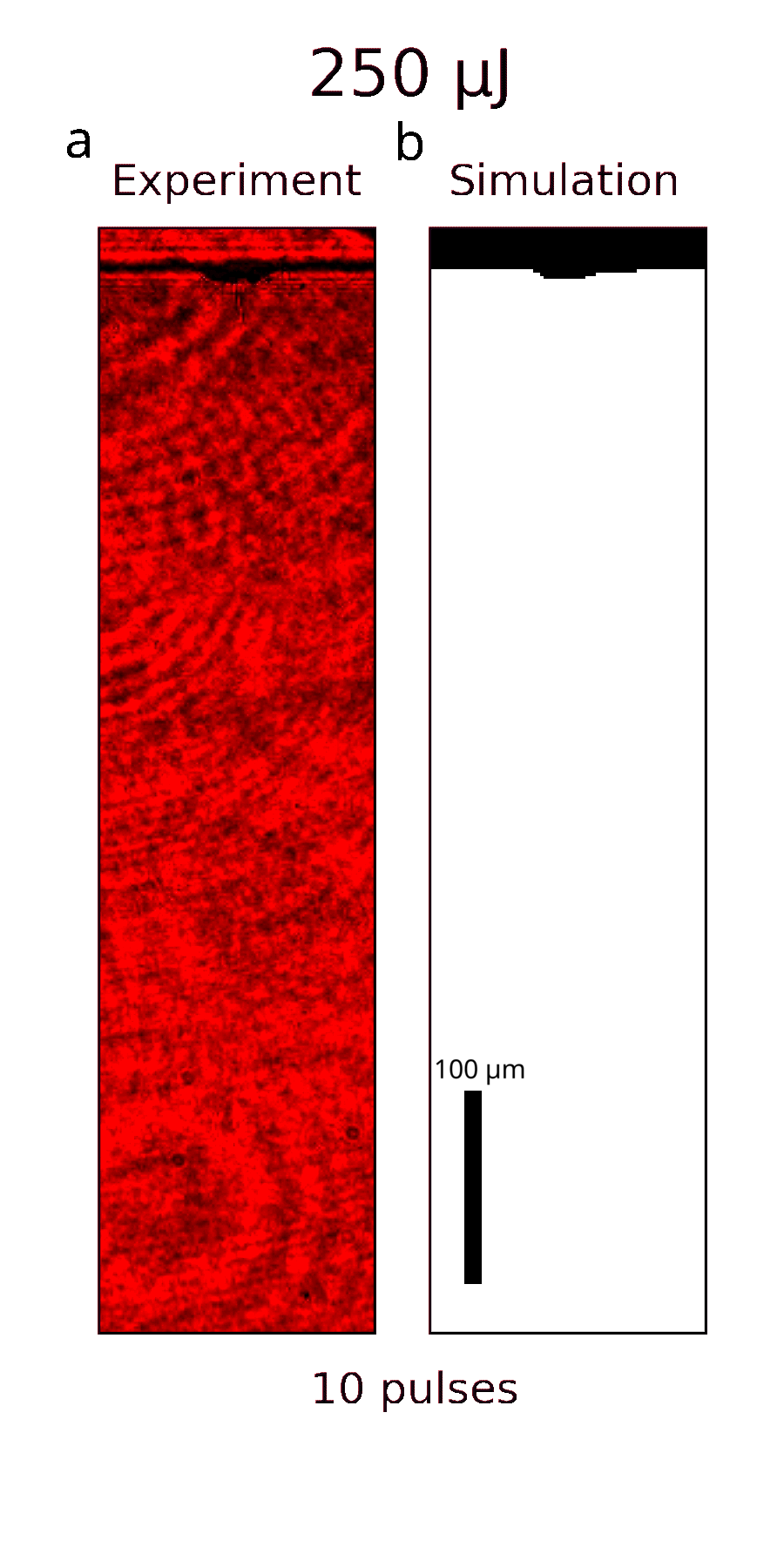

Supplement: Supplementary file 6 — Supplementary Movie 3 [file 44172_2022_48_MOESM6_ESM.gif]

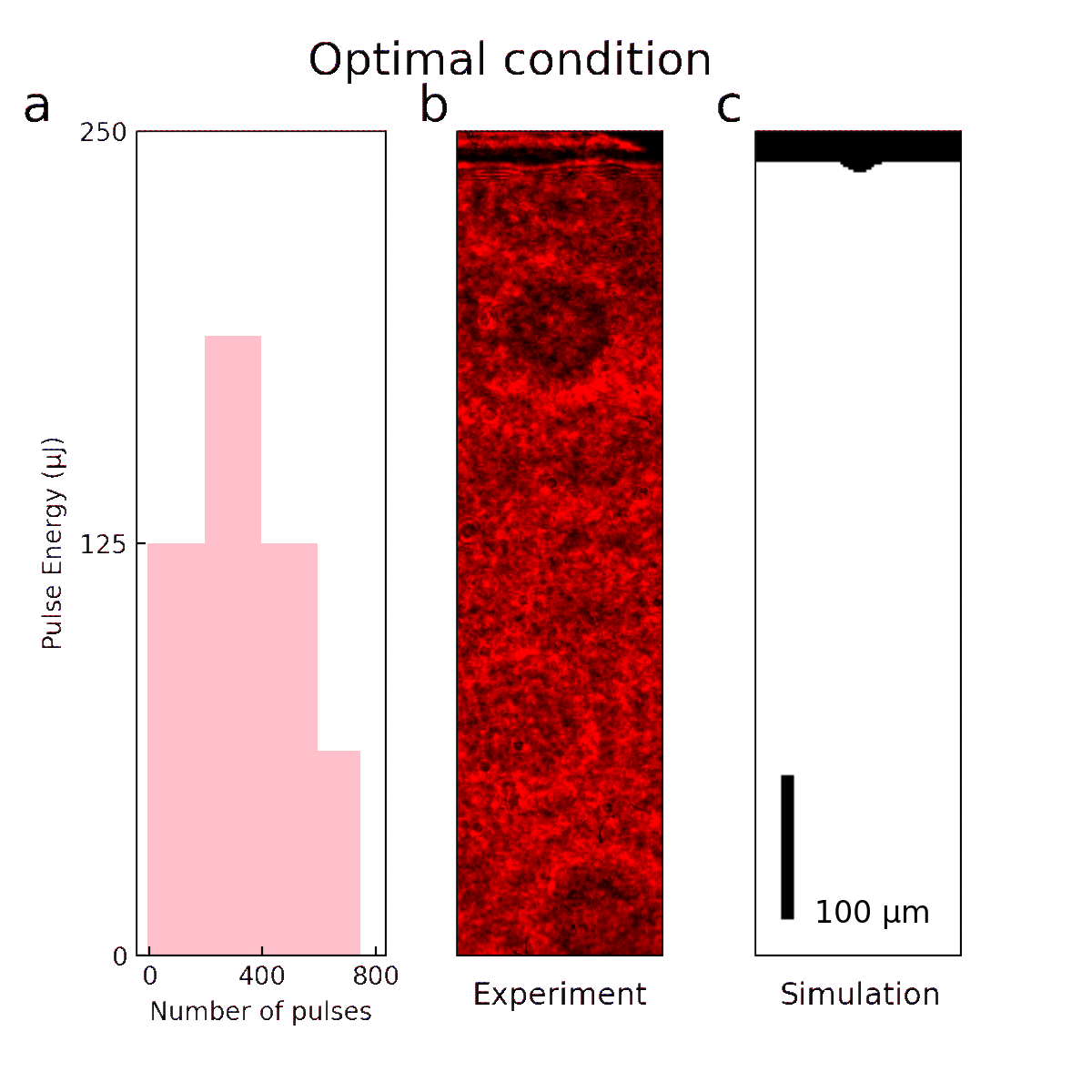

Supplement: Supplementary file 7 — Supplementary Movie 4 [file 44172_2022_48_MOESM7_ESM.gif]
